# Expression and Potential Prognostic Value of SOX9, MCL-1 and SPOCK1 in Gastric Adenocarcinoma

**DOI:** 10.3389/pore.2022.1610293

**Published:** 2022-02-09

**Authors:** Wenyi Luo, Teddy S. Nagaria, Hongxia Sun, Junsheng Ma, Jamie L. Lombardo, Roland Bassett, Austin C. Cao, Dongfeng Tan

**Affiliations:** ^1^ Department of Pathology, University of Oklahoma Health Sciences Center, Oklahoma City, OK, United States; ^2^ Department of Pathology and Laboratory Medicine, University of Texas MD Anderson Cancer Center, Houston, TX, United States; ^3^ Department of Pathology, McGill University, Montreal, QC, Canada; ^4^ Department of Pathology and Laboratory Medicine, University of Texas McGovern Medical School at Houston, Houston, TX, United States; ^5^ Department of Biostatistics, University of Texas MD Anderson Cancer Center, Houston, TX, United States; ^6^ Department of Pathology, Walter Reed National Military Medical Center, Bethesda, MD, United States; ^7^ Perelman School of Medicine, University of Pennsylvania, Philadelphia, PA, United States

**Keywords:** prognosis, gastric cancer, SPOCK1, SOX9, MCL-1

## Abstract

Gastric cancer is a common malignancy and remains one of the leading causes of cancer-related deaths, though its incidence is in decline in most developed countries. One of the major challenges of treating gastric cancer is tumor heterogeneity, which portends a high degree of prognostic variance and the necessity for different treatment modalities. Tumor heterogeneity is at least in part due to divergent differentiation of tumor cells to clones harboring different molecular alterations. Here we studied the expression of emerging prognostic markers SOX9, MCL-1, and SPOCK1 (Testican-1) in a cohort of gastric cancer by immunohistochemistry and investigated how individual biomarkers and their combinations predict disease prognosis. We found frequent expression of SPOCK1 (in both nuclei and cytoplasm), MCL-1 and SOX9 in gastric cancer. In univariate analysis, nuclear SPOCK1 expression and pathologic TNM stage were negative prognostic markers in this cohort. In multivariate analysis, SOX9 expression stood out as a predictor of poor prognosis. Further subgroup analysis suggested prognostic value of SOX9 expression in poorly differentiated gastric adenocarcinoma. MCL-1 showed no prognostic role in this cohort.

## Introduction

Gastric cancer is the fifth most common malignancy in the world and remains the third highest cause of cancer-related mortality after lung and colorectal cancer (1). The incidence of gastric cancer in young adults is increasing, and cancers in these patients are more aggressive and present at a more advanced stage (2).

The outcomes of gastric cancer patients depend on tumor resectability (3). Although the survival in patients after curative resection has improved from 14% in the 1980s to 49% in most recent series, forty to fifty percent of the gastric cancer patients recured (3,4). Eighty percent of gastric cancer patients present with unresectable and metastatic disease (4). The discovery of druggable molecular targets especially HER2/neu and HER2/neu monoclonal antibody therapy have significantly improved the median overall survival (5), suggesting the benefit of additional biomarker discovery.

The variable prognosis and response to treatment are partially dictated by the heterogeneity of the disease, which is associated with treatment resistance, non-uniform therapeutic effects, and clonal evolution of tumor cells (6). Although gastric cancer can be classified under four major molecular subtypes: Epstein–Barr virus, microsatellite instability, genomically stable, and chromosomal instability, based on predominantly expressed genes (7), the differences within each group are not negligible (8). Additional biomarkers or prognostic factors may be needed to further stratify the tumors in each major molecular subtype (8).

Here we present a study on the expression of emerging biomarkers SOX9, MCL-1 and SPOCK1 in a gastric cancer tissue microarray (TMA). Their correlation with differentiation markers, expression of PD-L1 and HER2/neu and prognosis was also explored.

## Materials and Methods

### Patients

The study included 238 consecutive primary gastric cancer patients who underwent surgical resection with no preoperative treatment at the University of Texas MD Anderson Cancer Center between 1987 and 2006. High-density tissue TMA was assembled from selected regions of archived formalin-fixed paraffin-embedded donor tissue containing viable tumor and normal tissue elements as described previouslyf (9). Clinicopathologic parameters collected in this study include age, race, gender, tumor differentiation (classified as well/moderately differentiated, poorly differentiated or signet ring cell carcinoma), pathologic TNM stage of the tumor and survival. The study was approved by institutional review board (IRB#: LAB07-0592).

### Immunohistochemistry

The expression of SOX9, SPOCK1 (Testican-1), MCL-1, MUC2, MUC5AC, CD10, PD-L1 and HER2/neu was assessed by immunohistochemical stains (Table 1).

**TABLE 1:** Antibody information.

Antibody	Manufacturer	Clone	Dilution
MCL-1	Cell Signaling	D5V5L	1:100
MUC2	Cell Marque	MRQ-18	1:300
MUC5AC	Dako	CLH2	1:50
SOX9	Millipore	AB5535	1:200
CD10	Leica	56C6	1:50
SPOCK1	Abcam	ab229935	1:500
HER2	Ventana	4B5	Premade
PD-L1	Dako	22C3	Premade

The immunoreactivity was quantified as described (10) using a weighted histoscore method based on intensity of staining as well as percentage of cell positivity. Thirty percent of total core number was scored by two observers (WL and HS) independently. The HER2/neu immunohistochemical stain was scored based on ASCO and CAP guidelines (11). PD-L1 was scored based on combined positive score with a cutoff of 1 or more (12).

Cases with incomplete data, including the cases whose corresponding tissue cores were lost during staining or cases with no tumor present in corresponding tissue cores, were excluded. Therefore, different number of cases were scored for each marker.

### Statistical Analysis

Categorical variables were summarized by frequencies and percentages and compared between groups with Fisher’s exact tests; continuous variables were summarized using medians and ranges, and assessed between groups by Wilcoxon rank sum tests or Kruskal-Wallis rank sum tests. Cox models and Firth penalized Cox models were used to evaluate the associations between survival outcome and covariates of interest.

## Results

### Patient and Tumor Characteristics

The majority of the patients were male Caucasians (59.7%). The median age at initial diagnosis was 64.1 years (range 28.4–89.6). Patient and tumor characteristics are summarized in Table 2.

**TABLE 2:** Patient and tumor characteristics.

Variable	Levels	n	%
Age	≤65	124	52.8
	>65	111	47.2
	all	235	100.0
Race	Asian	32	13.4
	Black	25	10.5
	Caucasian	142	59.7
	Hispanic	35	14.7
	Other	4	1.7
	all	238	100.0
Gender	Female	88	37
	Male	150	63
	all	238	100.0
Tumor differentiation	Poorly	85	36.8
	Signet ring cell	47	20.4
	Well/Moderate	99	42.9
	all	231	100.0
T stage	T1	54	22.7
	T2	25	10.5
	T3	106	44.5
	T4	53	22.3
	all	238	100.0
N stage	N0	63	28.1
	N1	48	21.4
	N2	53	23.7
	N3	60	26.8
	all	224	100
M stage	M0	205	86.1
	M1	33	13.9
	all	238	100.0

### Expression of Biomarkers in Gastric Cancer

**FIGURE 1:**
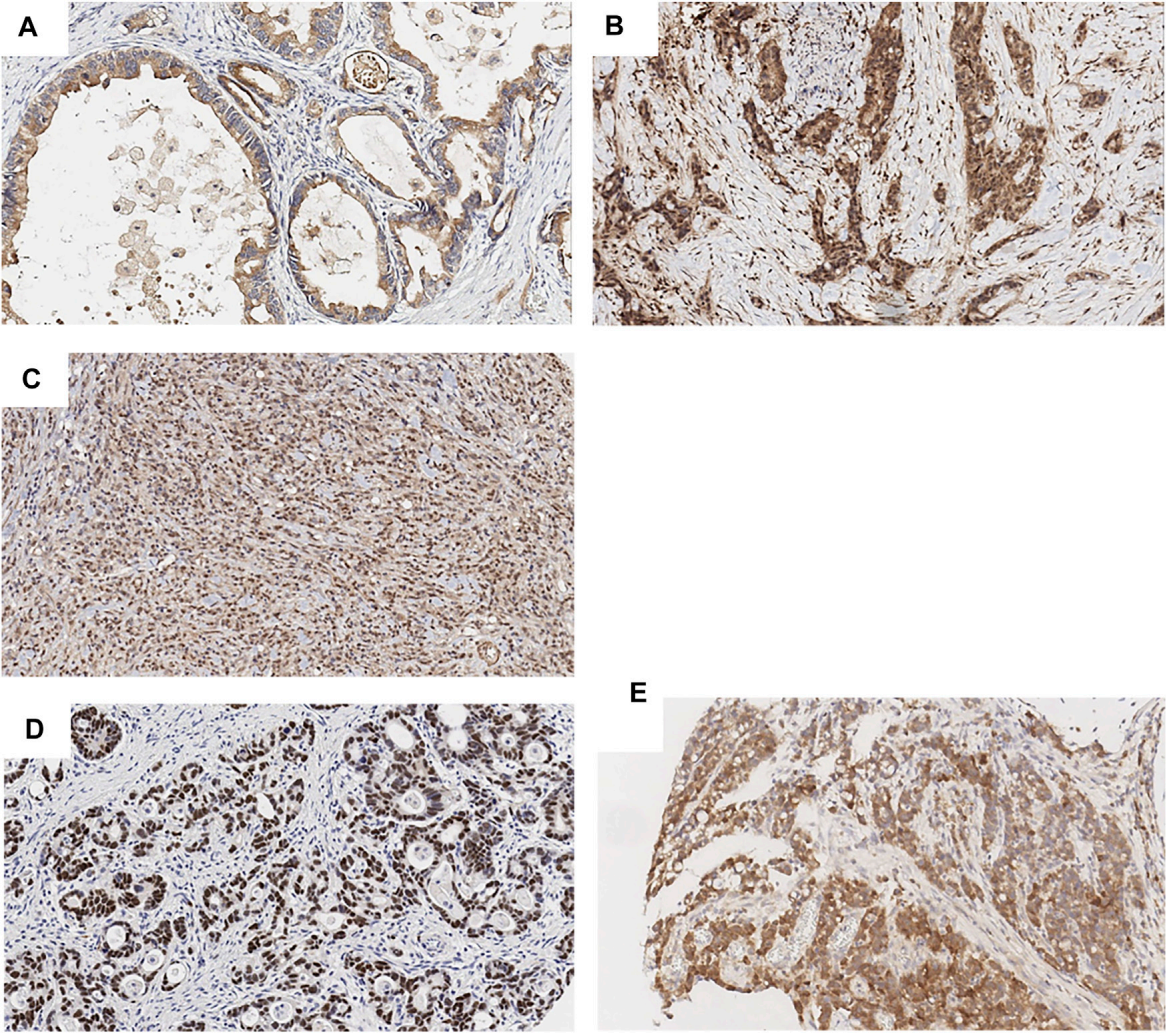
Biomarker expression in gastric cancer (by IHC, ×20). SPOCK1 apical **(A)**, cytoplasmic **(B)** and nuclear **(C)** expression. SOX9 nuclear expression **(D)**. MCL-1 cytoplasmic expression **(E)**.

The expression of SOX9, SPOCK1 (Testican-1), MCL-1 was assessed by immunohistochemical stains and correlated with differentiation markers MUC2, MUC5AC, CD10 as well as prognostic/predictive markers PD-L1 and HER2/neu. As previously reported, SOX9 showed nuclear stain (13) and MCL-1 showed cytoplasmic staining (14) (Figure 1). SPOCK1, however, showed three different patterns including apical (staining at the apical cell membrane of tumor cells), cytoplasmic and nuclear staining (Figure 1).

Based on how the scores are distributed, SOX9 expression was classified as high, moderated and low. MCL-1 and SPOCK1 expression was classified as high and low. Among 201 tumors scored for SOX9, 64 (31.8%) showed high expression, 62 (30.9%) showed moderate expression, and 75 (37.3%) showed low expression; Among 184 tumors scored for MCL-1, 93 tumors (51%) showed high expression; Among 144 tumors scored for SPOCK1, 78 tumors (54%) showed high nuclear expression, 107 tumors (74%) showed high cytoplasmic expression, and only 2 cases showed apical membranous expression.

### Correlations Between Biomarkers

SOX9 expression was positively correlated with MUC2 expression (*p* = 0.01) when SOX9 expression was categorized as low, moderated and high. When median was used, high SOX9 expression was associated with male gender (*p* = 0.036), negative PD-L1 (*p* = 0.034) and high SPOCK1 nuclear expression (*p* = 0.029). MCL-1 expression was associated with well and moderately differentiated tumors (*p* = 0.026) and lower T stage (*p* = 0.032). However, no correlation was discovered with other clinicopathologic parameters and other biomarker expression using either low, moderated and high expression or median.

### Prognostic Value of Biomarkers

The median survival time was 28.7 (21.8–42.2) months. Univariate analysis (Table 3) demonstrated that high pathologic TNM stage and nuclear SPOCK1 expression were associated with a worse overall survival, while Asian race was associated with better prognosis. We also fitted multivariate Cox models including covariates of race, M stage, tumor differentiation, SOX9, where these variables had *p*-values less than 0.25 in univariate analysis. As tumor stage of T, N and M are highly correlated, we only included M stage as it had more complete data. Other biomarkers or covariates with substantially small number of observations were not included. Multivariable analysis (Table 4) demonstrated that presence of metastasis (pM1) and SOX9 expression were predictors of poor prognosis, while better tumor differentiation was a predictor of good prognosis. In subgroup analyses (Table 4), the prognostic potential of SOX9 expression persisted only in patients with poorly differentiated tumors. MCL-1 was not associated with overall survival.

**TABLE 3:** Univariate analysis for overall survival.

Prognostic Factor	N	Events	HR	95% CI for HR	*p*
Age	231	186	1.00	(0.99, 1.01)	0.837
Race	227	186			
Caucasian (ref)	138	118	—	—	—
Asian	32	23	0.61	(0.39, 0.95)	**0.028**
Black	25	23	0.79	(0.50, 1.24)	0.309
Hispanic	32	22	0.67	(0.43, 1.07)	0.092
Gender	231	186			
Female (ref)	88	70	—	—	—
Male	143	116	1.05	(0.78, 1.41)	0.745
T stage	231	186			
T1 (ref)	53	27	—	—	—
T2	23	17	1.88	(1.02, 3.45)	**0.042**
T3	104	95	3.25	(2.11, 5.01)	**<0.001**
T4	51	47	4.09	(2.53, 6.61)	**<0.001**
N stage	217	174			
N0 (ref)	61	41	—	—	—
N1	47	36	1.22	(0.77, 1.92)	0.396
N2	52	46	2.25	(1.47, 3.45)	**<0.001**
N3	57	51	2.67	(1.76, 4.06)	**<0.001**
M.stage	231	186			
M0 (ref)	198	154	—	—	—
M1	33	32	4.24	(2.83, 6.36)	**<0.001**
Tumor differentiation	225	181			
Poor	83	67	—	—	—
Signet ring	46	41	1.3	(0.88, 1.92)	0.187
Well/Moderate	96	73	0.74	(0.53, 1.04)	0.079
MUC2	194	160	0.99	(0.91, 1.08)	0.894
MUC5AC	195	162	1.03	(0.98, 1.07)	0.253
SOX9	194	160	1.04	(0.98, 1.09)	0.206
MCL-1 Median	180	143			
0–39 (ref)	88	71	—	—	—
40–300	92	72	1.06	(0.76, 1.48)	0.735
SPOCK1 apical	139	117			
Low (ref)	128	108	—	—	—
High	11	9	0.88	(0.44, 1.74)	0.707
SPOCK1 cyto	139	117			
Low (ref)	35	25	—	—	—
High	104	92	1.49	(0.95, 2.32)	0.080
SPOCK1 nucleus	139	117			
Low (ref)	62	50	—	—	—
High	77	67	1.47	(1.00, 2.16)	**0.047**

Bold values are "significant P values".

**TABLE 4:** Multivariate survival analysis.

Variable	HR	95% CI for HR	*p*
**Multivariate analysis for overall survival in all tumors**			
Race			
Caucasian (ref)	—	—	—
Asian	0.64	(0.38, 1.08)	0.097
Black	0.91	(0.55, 1.45)	0.711
Hispanic	0.80	(0.47, 1.28)	0.361
M stage			
M0 (ref)	—	—	—
M1	4.23	(2.69, 6.65)	**<0.001**
Tumor differentiation			
Poorly (ref)	—	—	—
Signet ring	0.85	(0.55, 1.30)	0.458
Well/Moderate	0.58	(0.40, 0.83)	0.003
**SOX9**	1.08	(1.02, 1.14)	**0.011**
**Multivariate analysis for overall survival in poorly differentiated carcinoma**			
Race			
Caucasian (ref)	—	—	—
Asian	0.74	(0.32, 1.52)	0.426
Black	1.06	(0.49, 2.09)	0.875
Hispanic	1.06	(0.41, 2.32)	0.895
M stage			
M0 (ref)	—	—	—
M1	5.70	(2.84, 11.2)	**<0.001**
SOX9	1.1	(1.01, 1.19)	**0.033**

Bold values are "significant P values".

## Discussion

This study examined the expression and prognostic potentials of several emerging gastric cancer biomarkers. We found SOX9 was frequently expressed in gastric cancer. SOX9 is a transcription factor belonging to SOX (from *S*ry-related HMG b*ox*) family which is characterized by the presence of a conserved HMG DNA–binding domain. In embryogenesis and adulthood, SOX9 regulates cell fate decisions and stem cell maintenance including the gastrointestinal tract (15). SOX9 was found to be overexpressed in a wide range of human malignancies and its expression correlated with tumor aggressiveness (16). In human cancer, its target genes include genes promoting stemness, extracellular matrix, cell adhesion, cytoskeleton remodeling and invasion (17). SOX9-positive cells have characteristics of cancer stem cell. They are capable of self-renewal and differentiation to both SOX9-positive and SOX9-negative populations (18). SOX9-positive cells have high proliferation ability and are involved in epithelial-mesenchymal transition and chemotherapy resistance (18,19). Therefore, SOX9 is considered a cancer stem cell marker. SOX9 is involved in tumor initiation through Wnt/β-catenin pathway as well as tumor invasion through activation of TGFb/Smad signaling (17,18). Co-activation of NOTCH signaling pathway has also been identified (20). In normal human stomach, SOX9 is predominantly expressed in the neck/isthmus of the pyloric region with a small amount expressed in the neck/isthmus of the corpus (21). In mucosa with intestinal metaplasia, SOX9 expression is located at the base of the mucosa and colocalized in cells with the highest proliferation (21). SOX9 expression is significantly increased in *Helicobacter pylori*–positive gastric biopsies and its expression is required for bacteria-induced gastric cancer cell proliferation in a Wnt/β-catenin pathway dependent manner (19). Similar to our study, a prior study has also demonstrated SOX9 expression is common in gastric cancer (22). The correlation of SOX9 expression with tumor progression is controversial. A few studies suggested that elevated SOX9 expression was associated with either tumor aggressiveness or worse patient survival: One small scale study based on gastric cancer biopsies demonstrated high SOX9 expression was associated with more advanced tumor TNM stage and lower overall survival and disease-free survival (19); Another study only found correlation of SOX9 expression with TNM and clinical stages (23); Two other cohort studies did not find any significant relationship between altered expression of SOX9 protein and clinicopathological parameters including overall survival (24,25). On the contrary, one study found an inverse correlation between SOX9 expression and advanced tumor stage, vessel infiltration, nodal metastasis, and EBV infection (22). These contrasting findings may be attributed to varied roles of SOX9 in tumors of different degree of differentiations, tumor stages, patient populations, or dose-dependent effects. In this cohort, SOX9 expression was associated with poor prognosis especially in poorly differentiated tumors. More frequent SOX9 expression found in males might be a recapitulation of its preferential expression in male during the stage of sex development (26). The association of SOX9 expression with MUC2 (an intestinal differentiation marker) expression is consistent with its role in *Helicobacter pylori* infection and overrepresentation of intestinal-type histology in *Helicobacter pylori*-related gastric cancers (27). The negative association of SOX9 with PD-L1 suggests gastric cancer with “stem cell features” might be resistant to PD-L1-inhibitor-based immunotherapy.

The study discovered different expression patterns of SPOCK1 and frequent nuclear and cytoplasmic SPOCK1 expression in gastric cancer. SPOCK1 is a matricellular glycoprotein belonging to a novel Ca(2+)-binding proteoglycan family. Members of this protein family including SPOCK1 (TESTICAN-1), TESTICAN-2, TESTICAN-3 and SPARC share a similar N-terminus, follistatin-like domain, and C-terminus. They are involved in cell proliferation, adhesion, and migration (28). High tumoral SPOCK1 expression has been associated with increased growth, invasiveness and metastasis in many cancer types (29,30). Although the regulators of SPOCK1 vary in different cancer types, its tumor-promoting effects are predominantly through PI3K/Akt and Wnt/beta-catenin pathways (31). Stromal SPOCK may be important too because it modifies composition of stromal collagen and enables growth of pancreatic ductal adenocarcinoma in response to tumor-generated TGF-beta (32). The potential importance of SPOCK1 in gastric cancer was first raised in a genome-wide study that found SPOCK1 expression was upregulated 10-fold in gastric cancer (33). The elevated SPOCK1 expression has been associated with increased metastasis and a poor prognosis in patients with gastric cancer (34). SPOCK1 facilitates gastric cancer cell invasion and metastasis *in vitro* and mechanism appears to involve Slug-induced epithelial mesenchymal transition (34). In this cohort, the nuclear SPOCK1 expression correlated with a worse overall survival in univariate analysis. In addition, SPOCK1 expression correlated with SOX9 expression. Similar interaction has been identified in neuroblastoma in which significantly decreased SPOCK1 RNA levels were detected in SOX9 knockout neuroblastoma cells (35). These findings suggest potential interaction between two pathways.

Myeloid leukemia cell differentiation protein 1 (MCL-1) is an anti-apoptotic member of the BCL2 family proteins (36). MCL-1’s anti-apoptotic function is fulfilled by sequestering pro-apoptotic proteins BAK and BAX (37). In addition to anti-apoptosis, MCL-1 also promotes epithelial-mesenchymal transition of human cancer cells (38). In gastric cancer, MCL-1 expression has been correlated with poor prognosis (39) and resistance to chemotherapy (40). Suppression of MCL-1 produced a significant increase in apoptosis and up to 60% decrease in gastric cancer cell growth (41). The expression of MCL-1 in stomach is regulated by hypoxia-inducible factor 1 alpha (HNF1-α) in *Helicobacter pylori*-infected human gastric epithelium (42) and HMGB1 in gastric cancer cells (43). Our study did not confirm the prognostic value of MCL-1. We did find, however, that MCL-1 expression was associated with well and moderately differentiated tumors and lower T stage. These findings are different from prior studies. Since all the prior studies were published on Asian populations, the difference might be explained by different oncogenic etiologies. Alternatively, MCL-1’s activity, but not quantity, might be upregulated in poorly differentiated or high stage tumors by post-transcriptional regulation. Since IHC only measures protein quantity, it would not be able to identify the correlation between MCL-1 and tumor aggressiveness.

Our study was limited by drawbacks inherent to TMA such as nonrepresentative tumor sampling and staining artifacts (especially edge artifact). Further validation in full tissue sections is warranted.

## Data Availability

The original contributions presented in the study are included in the article/Supplementary Material, further inquiries can be directed to the corresponding author.

## References

[1] Rawla P Barsouk A Epidemiology of Gastric Cancer: Global Trends, Risk Factors and Prevention. pg (2019) 14:26–38. 10.5114/pg.2018.80001 PMC6444111 30944675

[2] Bergquist JR Leiting JL Habermann EB Cleary SP Kendrick ML Smoot RL Early-onset Gastric Cancer Is a Distinct Disease with Worrisome Trends and Oncogenic Features. Surgery (2019) 166:547–55. 10.1016/j.surg.2019.04.036 31331685

[3] D'Angelica M Gonen M Brennan MF Turnbull AD Bains M Karpeh MS Patterns of Initial Recurrence in Completely Resected Gastric Adenocarcinoma. Ann Surg (2004) 240:808–16. 10.1097/01.sla.0000143245.28656.15 15492562 PMC1356486

[4] Ajani JA Lee J Sano T Janjigian YY Fan D Song S Gastric Adenocarcinoma. Nat Rev Dis Primers (2017) 3:17036. 10.1038/nrdp.2017.36 28569272

[5] Bang Y-J Van Cutsem E Feyereislova A Chung HC Shen L Sawaki A Trastuzumab in Combination with Chemotherapy versus Chemotherapy Alone for Treatment of HER2-Positive Advanced Gastric or Gastro-Oesophageal junction Cancer (ToGA): a Phase 3, Open-Label, Randomised Controlled Trial. The Lancet (2010) 376:687–97. 10.1016/s0140-6736(10)61121-x 20728210

[6] Hudler P Challenges of Deciphering Gastric Cancer Heterogeneity. Wjg (2015) 21:10510–27. 10.3748/wjg.v21.i37.10510 26457012 PMC4588074

[7] Cancer Genome Atlas Research N. Comprehensive Molecular Characterization of Gastric Adenocarcinoma. Nature (2014) 513:202–9. 10.1038/nature13480 25079317 PMC4170219

[8] Lim B Kim JH Kim M Kin SY. Genomic and Epigenomic Heterogeneity in Molecular Subtypes of Gastric Cancer. Wjg (2016) 22:1190–201. 10.3748/wjg.v22.i3.1190 26811657 PMC4716030

[9] Truong CD Feng W Li W Khoury T Li Q Alrawi S Characteristics of Epstein-Barr Virus-Associated Gastric Cancer: a Study of 235 Cases at a Comprehensive Cancer center in U.S.A. J Exp Clin Cancer Res (2009) 28:14. 10.1186/1756-9966-28-14 19192297 PMC2642773

[10] Campbell KJ Dhayade S Ferrari N Sims AH Johnson E Mason SM MCL-1 Is a Prognostic Indicator and Drug Target in Breast Cancer. Cell Death Dis (2018) 9:19. 10.1038/s41419-017-0035-2 29339815 PMC5833338

[11] Bartley AN Washington MK Ventura CB Ismaila N Colasacco C Benson AB HER2Testing and Clinical Decision Making in Gastroesophageal Adenocarcinoma. Am J Clin Pathol (2016) 146:647–69. 10.1093/ajcp/aqw206 28077399 PMC6272805

[12] Kulangara K Zhang N Corigliano E Guerrero L Waldroup S Jaiswal D Clinical Utility of the Combined Positive Score for Programmed Death Ligand-1 Expression and the Approval of Pembrolizumab for Treatment of Gastric Cancer. Arch Pathol Lab Med (2019) 143:330–7. 10.5858/arpa.2018-0043-oa 30028179

[13] Mesquita P Freire AF Lopes N Gomes R Azevedo D Barros R Expression and Clinical Relevance of SOX9 in Gastric Cancer. Dis Markers (2019) 2019:8267021. 10.1155/2019/8267021 31275454 PMC6589301

[14] Lee W-S Park Y-L Kim N Oh H-H Son D-J Kim M-Y Myeloid Cell Leukemia-1 Regulates the Cell Growth and Predicts Prognosis in Gastric Cancer. Int J Oncol (2015) 46:2154–1262. 10.3892/ijo.2015.2890 25672320

[15] Kawaguchi Y Sox9 and Programming of Liver and Pancreatic Progenitors. J Clin Invest (2013) 123:1881–6. 10.1172/jci66022 23635786 PMC3635727

[16] Matheu A Collado M Wise C Manterola L Cekaite L Tye AJ Oncogenicity of the Developmental Transcription Factor Sox9. Cancer Res (2012) 72:1301–15. 10.1158/0008-5472.can-11-3660 22246670 PMC3378515

[17] Larsimont J-C Youssef KK Sánchez-Danés A Sukumaran V Defrance M Delatte B Sox9 Controls Self-Renewal of Oncogene Targeted Cells and Links Tumor Initiation and Invasion. Cell Stem Cell (2015) 17:60–73. 10.1016/j.stem.2015.05.008 26095047

[18] Kawai T Yasuchika K Ishii T Miyauchi Y Kojima H Yamaoka R SOX9 Is a Novel Cancer Stem Cell Marker Surrogated by Osteopontin in Human Hepatocellular Carcinoma. Sci Rep (2016) 6:30489. 10.1038/srep30489 27457505 PMC4960550

[19] Santos JC Carrasco-Garcia E Garcia-Puga M Aldaz P Montes M Fernandez-Reyes M SOX9 Elevation Acts with Canonical WNT Signaling to Drive Gastric Cancer Progression. Cancer Res (2016) 76:6735–46. 10.1158/0008-5472.can-16-1120 27569216

[20] Song S Maru DM Ajani JA Chan C-H Honjo S Lin H-K Loss of TGF-β Adaptor β2SP Activates Notch Signaling and SOX9 Expression in Esophageal Adenocarcinoma. Cancer Res (2013) 73:2159–69. 10.1158/0008-5472.can-12-1962 23536563 PMC3745222

[21] Sashikawa Kimura M Mutoh H Sugano K SOX9 Is Expressed in normal Stomach, Intestinal Metaplasia, and Gastric Carcinoma in Humans. J Gastroenterol (2011) 46:1292–9. 10.1007/s00535-011-0443-5 21861142

[22] Sun M Uozaki H Hino R Kunita A Shinozaki A Ushiku T SOX9 Expression and its Methylation Status in Gastric Cancer. Virchows Arch (2012) 460:271–9. 10.1007/s00428-012-1201-7 22331131

[23] Zhou C-J Guo J-Q Zhu K-X Zhang Q-H Pan C-R Xu W-H Elevated Expression of SOX9 Is Related with the Progression of Gastric Carcinoma. Diagn Cytopathol (2011) 39:105–9. 10.1002/dc.21348 20301211

[24] Choi YJ Song JH Yoon JH Choi WS Nam SW Lee JY Aberrant Expression of SOX9 Is Associated with Gastrokine 1 Inactivation in Gastric Cancers. Gastric Cancer (2014) 17:247–54. 10.1007/s10120-013-0277-3 23812904

[25] Zhang N Chai D Du H Li K Xie W Li X Expression of Reg IV and SOX9 and Their Correlation in Human Gastric Cancer. BMC Cancer (2018) 18:344. 10.1186/s12885-018-4285-x 29587675 PMC5870489

[26] Croft B Ohnesorg T Hewitt J Bowles J Quinn A Tan J Human Sex Reversal Is Caused by Duplication or Deletion of Core Enhancers Upstream of SOX9. Nat Commun (2018) 9:5319. 10.1038/s41467-018-07784-9 30552336 PMC6293998

[27] Díaz P Valenzuela Valderrama M Bravo J Quest AFG *Helicobacter pylori* and Gastric Cancer: Adaptive Cellular Mechanisms Involved in Disease Progression. Front Microbiol (2018) 9:5. 10.3389/fmicb.2018.00005 29403459 PMC5786524

[28] Bradshaw AD Sage EH SPARC, a Matricellular Protein that Functions in Cellular Differentiation and Tissue Response to Injury. J Clin Invest (2001) 107:1049–54. 10.1172/jci12939 11342565 PMC209289

[29] Wang T Liu X Tian Q Liang T Chang P. Reduced SPOCK1 Expression Inhibits Non-small Cell Lung Cancer Cell Proliferation and Migration through Wnt/β-Catenin Signaling. Eur Rev Med Pharmacol Sci (2018) 22:637–44. 10.26355/eurrev_201802_14288 29461591

[30] Zhang LQ Wang Y Zhang L. Effects of shRNA-Mediated Knockdown of SPOCK1 on Ovarian Cancer Growth and Metastasis. Cel Mol Biol (Noisy-le-grand) (2015) 61:102–9. 10.14715/cmb/2015.61.7.16 26638890

[31] Yang J Yang Q Yu J Li X Yu S Zhang X SPOCK1 Promotes the Proliferation, Migration and Invasion of Glioma Cells through PI3K/AKT and Wnt/β-Catenin Signaling Pathways. Oncol Rep (2016) 35:3566–76. 10.3892/or.2016.4757 27108836

[32] Veenstra VL Damhofer H Waasdorp C Steins A Kocher HM Medema JP Stromal SPOCK1 Supports Invasive Pancreatic Cancer Growth. Mol Oncol (2017) 11:1050–64. 10.1002/1878-0261.12073 28486750 PMC5537700

[33] Marimuthu A Jacob HK Jakharia A Subbannayya Y Keerthikumar S Kashyap MK Gene Expression Profiling of Gastric Cancer. J Proteomics Bioinform (2011) 4:74–82. 10.4172/jpb.1000170 27030788 PMC4809432

[34] Chen D Zhou H Liu G Zhao Y Cao G Liu Q. SPOCK1 Promotes the Invasion and Metastasis of Gastric Cancer through Slug-Induced Epithelial-Mesenchymal Transition. J Cel Mol Med (2018) 22:797–807. 10.1111/jcmm.13357 PMC5783867 28940639

[35] Mondal T Juvvuna PK Kirkeby A Mitra S Kosalai ST Traxler L Sense-Antisense lncRNA Pair Encoded by Locus 6p22.3 Determines Neuroblastoma Susceptibility via the USP36-CHD7-SOX9 Regulatory Axis. Cancer Cell (2018) 33:417–34. 10.1016/j.ccell.2018.01.020 29533783

[36] Kaufmann SH Karp JE Svingen PA Krajewski S Burke PJ Gore SD Elevated Expression of the Apoptotic Regulator Mcl-1 at the Time of Leukemic Relapse. Blood (1998) 91:991–1000. 10.1182/blood.v91.3.991.991_991_1000 9446661

[37] Willis SN Chen L Dewson G Wei A Naik E Fletcher JI Proapoptotic Bak Is Sequestered by Mcl-1 and Bcl-xL, but Not Bcl-2, until Displaced by BH3-Only Proteins. Genes Dev (2005) 19:1294–305. 10.1101/gad.1304105 15901672 PMC1142553

[38] Lee W-S Kim N Park Y-R Oh H-H Myung E Kim S-H Myeloid Cell Leukemia-1 Promotes Epithelial-Mesenchymal Transition of Human Gastric Cancer Cells. Oncol Rep (2015) 34:1011–6. 10.3892/or.2015.4040 26058661

[39] Maeta Y Tsujitani S Matsumoto S Yamaguchi K Tatebe S Kondo A Expression of Mcl-1 and P53 Proteins Predicts the Survival of Patients with T3 Gastric Carcinoma. Gastric Cancer (2004) 7:78–84. 10.1007/s10120-004-0272-9 15224193

[40] Hu C-J Wang B Tang B Chen B-j. Xiao Y-F Qin Y The FOXM1-Induced Resistance to Oxaliplatin Is Partially Mediated by its Novel Target Gene Mcl-1 in Gastric Cancer Cells. Biochim Biophys Acta (Bba) - Gene Regul Mech (2015) 1849:290–9. 10.1016/j.bbagrm.2014.11.008 25482013

[41] Wacheck V Cejka D Sieghart W Losert D Strommer S Crevenna R Mcl-1 Is a Relevant Molecular Target for Antisense Oligonucleotide Strategies in Gastric Cancer Cells. Cancer Biol Ther (2006) 5:1348–54. 10.4161/cbt.5.10.3224 16969094

[42] Bhattacharyya A Chattopadhyay R Hall EH Mebrahtu ST Ernst PB Crowe SE Mechanism of Hypoxia-Inducible Factor 1α-Mediated Mcl1 Regulation in Helicobacter Pylori-Infected Human Gastric Epithelium. Am J Physiology-Gastrointestinal Liver Physiol (2010) 299:G1177–G1186. 10.1152/ajpgi.00372.2010 PMC2993173 20829524

[43] Zhan Z Li Q Wu P Ye Y Tseng H-Y Zhang L Autophagy-mediated HMGB1 Release Antagonizes Apoptosis of Gastric Cancer Cells Induced by Vincristine via Transcriptional Regulation of Mcl-1. Autophagy (2012) 8:109–21. 10.4161/auto.8.1.18319 22108005

